# Tracheostomy complications in otorhinolaryngology are rare despite the critical airway

**DOI:** 10.1007/s00405-021-06707-7

**Published:** 2021-03-03

**Authors:** Johanna Ruohoalho, Guanyu Xin, Leif Bäck, Katri Aro, Laura Tapiovaara

**Affiliations:** Department of Otorhinolaryngology-Head and Neck Surgery, University of Helsinki and Helsinki University Hospital, PO Box 263, 00029 HUS Helsinki, Finland

**Keywords:** Tracheostomy, Mortality, Morbidity, ENT, Tracheotomy, Airway

## Abstract

**Purpose:**

To identify complications of surgical tracheostomies in otorhinolaryngologic patients and adjust our processes to be properly prepared in the future.

**Methods:**

We reviewed retrospectively all surgical tracheostomies (*n* = 255) performed by otolaryngologist-head and neck surgeons at Helsinki University Hospital between Jan 2014 and Feb 2017. Patient demographics, surgical details, surgical and medical complications, and tracheostomy-related mortality were recorded from the hospital charts. Risk factors for complications were assessed.

**Results:**

Altogether, 55 (22%) complications were identified in 39 (15%) patients, with pneumonia, accidental decannulation, and bleeding being the most common. No patient or surgery-related factor reached significance in overall complication risk factor analysis. Medical complications were more common after elective tracheostomies compared to emergency procedures (10.6% vs. 3.5%, *p* < 0.05). Majority of complications (78%) were classified as mild or moderate according to Clavien–Dindo. Only 2 (0.8%) tracheostomy-related deaths were recorded.

**Conclusion:**

In otorhinolaryngologists service, severe complications and tracheostomy-related deaths are very rare. Reducing their prevalence even further with careful planning is possible.

## Introduction

Tracheostomy is considered safe, whether it be traditional open surgical tracheostomy, or the newer percutaneous method [1]. However, some cases will ultimately terminate unfavourably, as even fatal complications occur [2, 3]. Despite being a widely used routine procedure with many indications, records on adult patients’ surgical tracheostomy-related complications remain limited. Otorhinolaryngologists—head and neck surgeons (ORL-HNS) perform a significant proportion of surgical tracheostomies [4], but data on complications in these are scarce. ORL-HNS treat patients with head and neck cancer (HNC), severe cervical infections and trauma. Therefore, they face the most critical airways and challenging tracheostomies, which may lead to a higher complication risk.

We retrospectively assessed all surgical tracheostomies performed by ORL-HNS in a Finnish tertiary care center between 2014 and 2017, with a focus on tracheostomy-related complications and mortality. The aim was to identify the types of problems that ORL-HNS may encounter and adjust our processes to be properly prepared in the future.

## Material and methods

In the present study, we collected data on all patients tracheostomized by ORL-HNS between January 2014 and February 2017 at the Helsinki University Hospital (HUS) district in Helsinki, Finland, covering approximately 1.6 million inhabitants. A total of 791 patients with NCSP (Nordic Classification of Surgical Procedures) codes GBB00 (tracheostomy) and GBA00 (tracheotomy) were identified. Generally, tracheostomy indicates a permanent stoma and tracheotomy is defined as a temporary opening of the trachea, but these terms are often used synonymously. Therefore, both codes were included. Out of these 791 patients, we manually screened every procedure that was performed by an ORL-HNS. Operations that were carried out by other than ORL-HNS (*n* = 514; 65.0%) were excluded from this study. All incorrectly coded operations (*n* = 5) and patients under 18 years of age (*n* = 17) were also excluded. Our study was approved by the Research Ethics Committee of the Helsinki University Hospital (DNRO 89/13/03/02C/2011; 13 Feb 2011), and an institutional study permission was granted (§10, February 6th, 30, 2017, HUS).

Patient demographics including age, sex, body mass index (BMI), physical status classification (American Society of Anesthesiologists score; ASA), and comorbidities (Adult Comorbidity Evaluation 27-score; ACE-27), were retrospectively reviewed from the hospital charts. Surgical details, including indication for tracheostomy, experience level of the operative surgeon, department, the type of anesthesia (local or general), type of surgery (elective or urgent), type of tracheal incision, other simultaneously performed surgeries, and time of decannulation, were also collected from hospital charts. Peri- and postoperative surgical site complications requiring inpatient care or surgical intervention and occurring within 12 months of the operation were screened. Medical complications within 30 days postoperatively were recorded. Complications were classified by severity according to the Clavien–Dindo classification [7]. Timing of the complication in relation to surgery was recorded and tracheostomy-related deaths were evaluated.

Statistical analyses were performed with SPSS software (IBM SPSS Statistics Version 25, Armonk, NY, USA). Risk factors for complications were screened using Pearson chi-squared test and logistic regression analysis. *P *values are two-sided, and the significance threshold is set at 0.05.

## Results

### Demographics

Table 1 summarizes the demographics of our patients. The final study group comprised 255 patients, predominantly male (71%), with a median age of 65 years (range 19–92). After the 12-month follow-up, 36% of patients remained tracheostomized. Cannulation period was 1–7 days in 27% of the patients, 8–30 days in 23%, and 31–319 days in 14% of the patients.

**Table 1:** Patient characteristics and operation-related factors (*n* = 255; %)

Sex	
Male	181 (71.0)
Female	74 (29.0)
Age, median (range)	65 (19–92)
BMI^a^, median (range)	23.9 (12–44)
ASA^a^	
1–2	74 (29.0)
3	116 (45.5)
4	57 (22.4)
5	5 (2.0)
Indication	
Head and neck cancer	190 (74.5)
Infection	32 (12.5)
Bilateral vocal cord paralysis	17 (6.7)
Prolonged intubation or intubation failure	8 (3.1)
Trauma	2 (0.8)
Other	6 (2.4)
Other simultaneous procedure	
Yes	191 (74.9)
No	64 (25.1)
Urgency of the operation	
Elective	141 (55.3)
Urgent	114 (44.7)
Anesthesia	
Local	172 (67.5)
General	83 (32.5)
Surgeon education level	
Specialist	160 (62.5)
Resident supervised by a specialist	95 (37.3)

^a^*BMI* Body mass index, *ASA* American Society of Anesthesiologists Physical Status Classification

Altogether, 198 (78%) patients had HNC. Most patients had an ASA classification of 3 or above (*n* = 178; 70%), which implies an overall impaired physical condition. Most of the patients, however, did not have many comorbidities, as the most common ACE-27 scores were 0 (*n* = 95; 37%) or 1 (*n* = 76; 30%) (median 1, range 0–3).

The type of tracheal incision could be assigned in 240 (94%) cases and most of them were made horizontally (*n* = 219; 91%).

### Complications

A total of 55 (22%) complications were detected in 39 (15%) patients. Eight of the patients had two, and four of the patients had three distinct complication events. The complications were further divided into site-specific and medical complications (Table 2). The most common surgical site complication was accidental decannulation (*n* = 10; 4%), which in two cases occurred during the first postoperative week. Local bleeding was reported in eight patients (3%), and in five cases (63%), it occurred on the day of operation or on the first postoperative day. The most common medical complication was pneumonia (*n* = 12; 5%). Timing of the complications is presented in Table 3.

**Table 2:** Complications of tracheostomy in 255 patients in total (%)

Surgical complications	
Accidental decannulation	10 (3.9)
Local bleeding	8 (3.1)
Tube blockage	3 (1.2)
Major infection	3 (1.2)
Delayed stoma closure	3 (1.2)
Subcutaneous emphysema	2 (0.8)
Pneumothorax	1 (0.4)
Cuff rupture	1 (0.4)
Loss of airway during procedure	1 (0.4)
Medical complications	
Pneumonia	12 (4.7)
Cerebrovascular event	3 (1.2)
Delirium	6 (2.4)
Sepsis	1 (0.4)
ARDS^a^	1 (0.4)

^*a*^*ARDS *Acute respiratory distress syndrome

**Table 3:** Timing and Clavien-Dindo classification of complications (*n* = 55; %)

Timing in relation to surgery	
Intraoperative	1 (1.8)
< 7 days postoperatively	32 (58.2)
7–30 days postoperatively	16 (29.1)
> 30 days postoperatively	6 (10.9)
Classification of complications by Clavien–Dindo	
I	2 (3.6)
II	20 (36.4)
IIIa	22 (40.0)
IIIb	1 (1.8)
IVa	7 (12.7)
IVb	0
V	3 (5.5)

Complication rate in specialists’ tracheostomies was 24%, and that of resident surgeons’ tracheostomies 17% (*p* = 0.34). Tendency toward elevated risk of medical complications in HNC patients was seen, but HNC status did not influence the occurrence of surgical complications (Table 4). Medical complications were more common after elective tracheostomies (10.6% vs 3.5%, *p* < 0.05). However, the urgency of the tracheostomy strongly correlated with patients’ HNC status and further with other simultaneous procedures. In elective group, almost all patients had HNC while in emergency group, the proportion of HNC patients was significantly lower (97% vs. 54%, *p* < 0.001). Correspondingly, simultaneous other procedures were performed more frequently in elective group than in emergency group (92% vs. 54%, *p* < 0.001).

**Table 4:** Occurrence of surgical and medical complications after tracheostomy according to patients HNC status and urgency of operation (%)

	HNC^a^ (*n* = 198)	Non-HNC^a^ (*n* = 57)	*p*	Elective (*n* = 141)	Urgent (*n* = 114)	*p*
Surgical complications	24 (12.1)	8 (14.0)	0.70	16 (11.3)	16 (14.0)	0.52
Medical complications	17 (8.6)	2 (3.5)	0.20	15 (10.6)	4 (3.5)	< 0.05

^a^*HNC *head and neck cancer

Furthermore, we evaluated the role of age, sex, tobacco consumption, BMI, ASA, and type of tracheal incision as complication risk factors, but no patient or surgery-related parameter could be recognized to increase the overall complication risk.

Complications were mostly mild or moderate, as 78% of complications were classified to Clavien–Dindo grades II–III (Table 3). Three patients (1.2%) deceased due to postoperative complications (Clavien–Dindo grade V), and two of the deaths were directly related to tracheostomy, indicating procedure-related mortality of 0.8%. One patient with recurrent HNC had challenging anatomy due to disease itself and post-treatment status, ensuing in perioperative airway loss, resuscitation, anoxic brain damage, and ultimately death five days later. Other tracheostomy-related death was associated with patient selection. The patient was tracheostomized due to bilateral vocal cord paralysis caused by neurological disease. Postoperatively, he was agitated and repetitively removed the tracheostomy tube. Two weeks postoperatively, attempts to reinsert the tube failed resulting in death of the patient. The third Clavien–Dindo Class V complication patient suffering from ARDS died on the 14th postoperative day, but he underwent major HNC surgery simultaneously with tracheostomy, and the respiratory system failure cannot be considered directly related to tracheostomy.

## Discussion

Despite the challenging airway ORL-HNS operate on, we found only scarce major complications in surgical tracheostomies, with 0.8% procedure-related mortality rate. However, mild deviations in the recovery were recorded in almost every sixth patient. Statistically significant risk factors for tracheostomy complications could not be recognized.

We report a total complication rate of 22%, which is less than some previous studies report, despite we recorded also medical complications. Lee et al [5] reported tracheostomy complication rate of 43% in patients with major oral cavity cancer resection. However, they did not report medical complications. Goezt et al [6] studied oral cancer patients operated with microvascular reconstruction, and revealed 20% tracheostomy complication rate, with pneumonia being the most common. Their result is comparable to ours. Publications addressing the medical complications of tracheostomies are limited. However, we wanted to include medical complications as well, although the causality between the tracheostomy procedure and a complication might be controversial. In our study, elective patients experienced medical complications more often compared to emergency group. Almost all patients in elective group had HNC, and majority of them underwent other simultaneous procedures with the tracheostomy. Mechanical ventilation and prolonged ICU treatment has been shown to increase the risk of postoperative complications including pneumonia after major head and neck surgery [7]. Thus, the medical complications in our study are probably largely related to simultaneous major tumor surgery, often followed by postoperative ICU treatment.

The most frequent complications in our study included pneumonia, accidental decannulation, and local bleeding. Bleeding is reported to be among the most common complications in many former studies [4, 8–11], as is decannulation [8, 12], tube obstruction [8, 12, 13], and stomal infection [12, 14]. We recorded only major infections, which can explain our low rate, comparing to, for example, 24% reported by Gilyoma et al [14]. However, the definition of a stomal infection is not clear and unfortunately not usually specified in publications. We recorded a stomal infection only if it required readmission to the hospital or operative treatment. As the nature of the procedure involves airway to be connected with skin, some degree of bacterial colonization is always present and the line between an infection and normal recovery is thereby vague.

Tracheal stenosis [4, 11] and suprastomal granulation tissue [13, 14] were common complications in certain studies, but neither was reported in our patient material. Tracheal stenosis is a relatively uncommon late complication that only arises after decannulation when the tracheal stoma has closed. In our hospital, the tracheal stoma is closed with sutures at the time of decannulation, which reduces the risk of tracheal stenosis [15]. Further, the most common tracheal incision used in our population was a simple horizontal cut, where damage to tracheal cartilage is minimal. Whether this reduces the risk of late tracheal stenosis remains uncertain and warrants further studies. Furthermore, our late complications were reported only if a patient had a complaint and contacted the clinic, leaving the cases of asymptomatic stenosis unrecorded.

There were only two deaths associated with tracheostomy among our 255 cases, which indicates surgery-related mortality < 1%. Our finding is consistent with the result of Halum et al [4] with 0.85% mortality. In addition, only one intraoperative complication leading to patient’s death was recorded in our series, which is comparable to described by Klemm et al [16] (0.62%). These numbers could be considered relatively low, particularly when taking into account the premise of these operations where airway may be critical.

We believe that the two procedure-related deaths might have been avoided by better planning. The first patient was known to have a difficult airway, but did not suffer from difficulties in breathing until the procedure was executed. Sometimes awake fiberoptic intubation is a good choice for securing the airway before performing elective tracheostomy, especially when one might encounter problems in the procedure. The other patient removed the cannula repeatedly, leading to loss of airway. With agitated, restless patients, we nowadays use sutures to assure that the cannula cannot be accidentally removed. These procedures are easily executed to enhance safety in tracheostomy.

ORL-HNS are familiar with the anatomy of upper airways, which is an important underlying factor for reducing the incidence of possible complications. In our material, only 35% of all tracheostomies identified in database search were operated by ORL-HNS. This relatively low rate can result from the distinct location of the Department of Otorhinolaryngology-Head and Neck Surgery in HUS district. Different departments are scattered in separate locations, and for example trauma patients and major burns are treated elsewhere, where ORL-HNS are not present. We believe that ORL-HNS experience in evaluating the airway is unique and should be utilized more, in particular for avoiding unnecessary tracheostomies and consulting on a known difficult airway. In Halum’s multi-institutional study on tracheostomies, ORL-HNS performed 66% of all procedures [4].

Our study has some limitations. The data collection was carried out retrospectively, and we did not contact the patients in search for long-term complications. Thus, some patients with minor and/or long-term complications might not be recorded in our database. In addition, we recorded our tracheostomies by searching corresponding NCSP codes for procedures, and some incorrectly coded patients might be excluded from this study. As complications were rare, our sample size was not large enough to perform a statistically valid analysis on the risk factors of any single complication. The strength of the study is a large number of patients recorded from a single University hospital district using the same patient chart and coding system. Further, all the data were collected and coded by a single person, which makes it more consistent and reliable.

## Conclusion

The most common complications of tracheostomy at our institution were pneumonia, accidental decannulation, and bleeding. Even though ORL-HNS perform the most challenging tracheostomies, severe complications and tracheostomy-related deaths are very rare. Minor complications occur, but the benefits of performing tracheostomy will predominantly outweigh the risks involved. With precise planning, together with the operative team and the anesthesiologist, even more complications could be avoided.

## Data Availability

Available from corresponding author for 10 years.
